# Many Faces of the Hidden Souls: Medical and Neurological Complications and Comorbidities in Disorders of Consciousness

**DOI:** 10.3390/brainsci11050608

**Published:** 2021-05-10

**Authors:** Bei Zhang, Karen Huang, Jay Karri, Katherine O’Brien, Craig DiTommaso, Sheng Li

**Affiliations:** 1 Department of Physical Medicine and Rehabilitation, McGovern Medical School, University of Texas Health Science Center at Houston, Houston, TX 77030, USA; Bei.Zhang@uth.tmc.edu (B.Z.); Karen.Huang@uth.tmc.edu (K.H.); 2 TIRR Disorders of Consciousness Program, TIRR Memorial Hermann Hospital, Houston, TX 77030, USA; Jay.Karri@bcm.edu (J.K.);; 3 H. Ben Taub Department of Physical Medicine and Rehabilitation, Baylor College of Medicine, Houston, TX 77030, USA; 4 US Physiatry, Houston, TX 77009, USA; craig.ditommaso@gmail.com

**Keywords:** disorders of consciousness, complications, comorbidities

## Abstract

Early and goal-directed management of complications and comorbidities is imperative to facilitate neurorecovery and to optimize outcomes of disorders of consciousness (DoC). This is the first large retrospective cohort study on the primary medical and neurological complications and comorbidities in persons with DoC. A total of 146 patients admitted to a specialized inpatient DoC rehabilitation program from 1 January 2014 to 31 October 2018 were included. The incidences of those conditions since their initial brain injuries were reviewed per documentation. They were categorized into reversible causes of DoC, confounders and mimics, and other medical/neurological conditions. The common complications and comorbidities included pneumonia (73.3%), pain (75.3%), pressure ulcers (70.5%), oral and limb apraxia (67.1%), urinary tract infection (69.2%), and 4-limb spasticity (52.7%). Reversible causes of DoC occurred very commonly. Conditions that may confound the diagnosis of DoC occurred at surprisingly high rates. Conditions that may be a source of pain occurred not infrequently. Among those that may diminish or confound the level of consciousness, 4.8 ± 2.0 conditions were identified per patient. In conclusion, high rates of various complications and comorbidities occurred in persons with DoC. Correcting reversible causes, identifying confounders and mimics, and managing general consequences need to be seriously considered in clinical practice.

## 1. Introduction

Severe and extensive injury to the brain may result in various degrees of alteration in the level of consciousness [1]. Patients with disorders of consciousness (DoC) are clinically classified along a spectrum of severity ranging from coma to minimally conscious state (MCS) [1]. This patient population is often immobile and unable to communicate, which makes them particularly susceptible to various medical and neurological complications and comorbidities. Some of these conditions are suggested to suppress and mask the expression of consciousness. This phenomenon may be associated with a high rate of misdiagnosis (up to 40% [2]).

Currently, there does not exist any well-established epidemiological data for medical and neurological complications and comorbidities in the DoC population. Several articles have attempted to investigate those conditions [3,4,5] but focused on the correlation with outcome prediction [4,5,6]. There remains concern that the information pertaining to clinical evaluation and management of these complications and comorbidities may be underrepresented. Conditions that are uncommon but easy to identify in the general brain injury population may become common and difficult to identify in the DoC population. Having the information is essential in assisting accurate clinical assessment and successful management across multiple specialties involved in the care of these patients. This study aims at reporting the primary medical and neurological complications and comorbidities from a clinical practice perspective in a specialized DoC rehabilitation program.

## 2. Materials and Methods

This is a retrospective review study based on existing documentations in the electronic medical record (EMR) of all patients admitted to a specialized DoC rehabilitation program in a free-standing rehabilitation hospital from 1 January 2014 to 31 October 2018. A pre-admission screen of the patients referred to as “DoC” was performed by the medical director and an experienced neuropsychologist by reviewing the patient’s medical record, talking with the referring liaison, and talking with the patient’s family, in order to determine the appropriateness of the admission to the specialized DoC program (triage potential misdiagnosis of DoC, for example, patients with clear evidence of global aphasia, patients requiring limb restraints or a sitter, patients referred early after a unilateral hemispheric injury, etc., who would be admitted to other brain injury rehabilitation services in the same facility). The requirements of being off the ventilation support and achieving spontaneous eye-opening (i.e., out of coma) were waived when it was found that a patient without spontaneous eye-opening due to bilateral 3rd cranial nerve injury was misdiagnosed as comatose, and some patients could be easily weaned off mechanical ventilation but have not due to concerns of their responsiveness. Other admission criteria included being medically stable for the transfer. Relevant demographic information, including etiology, age at the time of injury, gender, time since injury, etc., was obtained as described in a previously published paper [7].

Complications and comorbidities were categorized from a clinical practice perspective pertinent to the evaluation and management of DoC [1]. Reversible causes refer to conditions that may alter arousal and awareness. Confounders are conditions affecting the assessment of the level of consciousness; mimics are conditions with manifestations similar to but clinically distinct from DoC so they can be mistaken as DoC; but both of them do not typically affect the level of consciousness [1]. Therefore, the categories include (1) reversible causes of DoC, (2) confounders and mimics of DoC, and (3) other medical/neurological conditions. Complications and comorbidities were incidences that occurred at any point since their initial brain injuries, e.g., presented as a history, existed on admission, or occurred during the rehabilitation course, based on documentation. The incidence of an event was obtained by using the “Chart Search” function in the EMR with pertinent key words and/or direct lab results or diagnostic reports. Each incidence was only counted once in one individual, regardless of recurrence. The detailed chart search, review, and data collection strategy are reported in Supplement Table S1. In certain scenarios, the denominators were adopted from specific populations to ensure a more accurate calculation of the incidence rate, for example, spine fracture in traumatic cases. Clinical determination of emergence from minimally conscious state was made with a series of standardized assessments, including but not limited to Coma Recovery Scale—Revised, and close clinical observations by a dedicated group of interdisciplinary professionals with extensive experience of DoC. The study was approved by the local institutional review board.

## 3. Results

A total of 146 patients were reviewed. The majority of the patients were young (approximately 30 years of age), male (74.0%), and admitted within 1 month after injury (48.6%); 59.6% of whom had a traumatic brain injury (TBI), followed by 28.8% anoxic brain injury (ABI); as described before [7] and shown in Table 1.

A total of 43 conditions (marked with asterisks) are summarized in Table 2, Table 3 and Table 4. Collectively, the most common complications and comorbidities included feeding tube placement (100.0%), tracheostomy tube placement (97.9%), pneumonia (73.3%), pain (subjectively reported by 75.3% of the emerged patients), pressure ulcers (70.5%), oral and limb apraxia (suspected in 67.1% of the emerged patients), urinary tract infection (69.2%), spasticity in four limbs (52.7%), paroxysmal sympathetic hyperactivity (PSH, 47.9%), hydrocephalus requiring ventriculoperitoneal shunt placement (38.4%), seizure/seizure-like activity on EEG (29.5%), keratitis (26.0%), deep venous thrombosis (26.0%), cortical visual impairment (19.2%), ileus (19.2%), aphasia (suspected in 17.6% of the emerged patients), low testosterone in male patients (16.7%), nephrolithiasis (15.8%), and heterotopic ossification (HO, 15.8%).

Among the reversible causes of DoC, hydrocephalus, seizure, and infections occurred very commonly (>30%, Table 2). The usually overlooked and rarely reported but may be clinically important conditions included apraxia, aphasia, profound sensory deficits (e.g., deafness and blindness, spinal cord injury), diffuse motoric deficits (e.g., critical illness myopathy/neuropathy, spasticity), that may confound the evaluation and diagnosis of DoC, occurring at surprisingly high rates (Table 3). Conditions that mimic DoC were locked-in syndrome, catatonia, and akinetic mutism, occurring at a low rate (<5%), but can be seen (Table 3). Conditions, that may not usually be life-threatening or cause altered consciousness but may be a source of pain, included keratitis, ileus, HO, kidney stone, ingrown toenail, ulcer, etc., occurring not infrequently (Table 4).

In the full cohort, 10.4 ± 3.1 conditions occurred per patient. Among the first 21 conditions (Table 2 and Table 3) that may diminish or confound the level of consciousness, 4.8 ± 2.0 conditions occurred per patient (mean ± SD as both data followed normal distribution). The occurrence and distribution patterns were similar between traumatic and anoxic injury-related DoC.

## 4. Discussion

This study demonstrated high rates of various complications and comorbidities in persons with DoC, many of which are reported for the first time in this population. The conditions were categorized and summarized in a clinically practical scheme [1]. This scheme provides a meaningful perspective in interpreting the data and could function as a working sheet of differential diagnosis in practice. It lays out a new mindset helpful in the evaluation and management of these clinically complex patients.

As shown in the results, a patient with DoC is likely to have multiple complications and comorbidities. In previous studies, patients with more complications were at higher risks of poorer functional outcomes [3,5]. The results support the need for higher vigilance and more meticulous care from specialized services as proposed by the AAN/ACRM/NIDILRR DoC guidelines [8,9]. Recognizing and treating many of these conditions may be of paramount value in discovering the buried minds and minimizing covert suffering. Some reversible causes are not difficult to recognize and treat (e.g., metabolic abnormalities and infections), while others are. Making the diagnosis of hydrocephalus can be arduous in the setting of ventriculomegaly due to cerebral atrophy after a severe brain injury [10]. The behavioral evidence of seizure can be obscured by the presence of myoclonus and roving eye movements at baseline. Furthermore, non-convulsive status epilepticus has no observable behavioral evidence. For confounders of DoC (Table 3 conditions 1–6), diagnosing some of them (e.g., apraxia and aphasia) can be challenging due to limited understanding of the neural substrates and lack of effective tools. Otherwise, severe and diffused spasticity is a common confounder that needs to be well managed to facilitate voluntary movements for patients with DoC [7]. Although the mimics (Table 3 conditions 7–9) are considered distinct clinical entities, they are possible sequelae resulting from a severe brain injury that may be commonly overlooked and mistaken as DoC. Keeping those conditions in mind may prompt assessments (e.g., initiating a lorazepam trial for suspected catatonia) as they are usually known a posteriori. The concurrence of multiple abovementioned conditions will significantly limit linguistic and motoric responses and further complicate the assessment. Treating other conditions (Table 4) may not have direct impact on the level of consciousness, however, it can prevent subsequent serious consequences. For example, constipation and ileus may cause vomiting, thus increasing the risk of aspiration pneumonia. We therefore advocate for proactive interdisciplinary endeavors and actions to assess and manage these complications and comorbidities, as presented in Table 2, Table 3 and Table 4, in clinical practice.

It is important to point out that patients with DoC are susceptible to iatrogenic drug-induced sedation, potentially even with those generally considered least cognitive-limiting agents (e.g., antispasmolytics [7]). Modalities with minimal systemic effects or focal treatments would be preferred. Sometimes, managing some of the conditions (e.g., PSH and pain) could be fine art of trading off ideal stimulation for adequate comfort. This is better pursued after confirming diagnostic accuracy of the level of consciousness; otherwise, re-evaluated periodically.

The results were derived from real-world clinical practice aiming at best possible care and outcome for persons with DoC. The results were limited by the retrospective nature of data acquisition. More conditions may worth investigating (e.g., sleep disturbances, bilateral cranial nerve III palsies, etc.). Selection and subjective bias may exist in an uncontrolled, single-center clinical environment. Otherwise, the time course and concurrency of these conditions varied significantly. It was difficult to demonstrate their causal impact on the level of consciousness and recovery. However, general presumptions, which are usually negative, may be made based on the existing evidence [3,5,6]. A standardized tool, the Comorbidities Coma Scale (CoCoS), may be helpful in practice and future studies [11].

## 5. Conclusions

High rates of various medical and neurological complications and comorbidities occurred in persons with DoC. Correcting reversible causes, identifying confounders and mimics, and managing general consequences need to be seriously considered in clinical practice.

## Figures and Tables

**Table 1:** Demographics of the 146 patients.

Age (years old, mean ± SD)	36 ± 15
Gender (male, %)	108 (74.0%)
Etiology (*N*, %)	
TBI	87 (59.6%)
ABI	42 (28.8%)
Stroke	11 (7.5%)
Mixed	6 (4.1%)
Months since injury (*N*, %)	Mode: 1 Median: 2 [1,4] Mean: 8
≤1	71 (48.6%)
2–5	41 (28.1%)
≥6	34 (23.3%)
Diagnosis on admission (*N*, %)	
UWS/VS	63 (43.1%)
MCS	74 (50.7%)
Emerged	9 (6.2%)

TBI: traumatic brain injury; ABI: anoxic brain injury; UWS/VS: unresponsive wakefulness syndrome/vegetative state; MCS: minimally conscious state.

**Table 2:** Common complications and comorbidities that can be reversible causes of DoC.

Conditions	Number of Cases (*N* = 146, Unless Noted Otherwise)		Incidence Rate
	Yes	No	
**1. Hydrocephalus status**			
(1) Hydrocephalus s/p VPS *	56	90	38.4% (56/146)
(2) CT head findings		(No CT head: 1)	
Hydrocephalus	57 (VPS: 52)	/	
Ventriculomegaly	57 (VPS: 4)	/	
No hydrocephalus	31	/	
(3) VPS malfunction/revision	27	/	48.2% (27/56)
**2. Seizure**			
(1) Physical observations		(No documented concern: 59)	
History	37	/	25.3% (37/146)
Tonic-clonic	17	/	11.6% (17/146)
Twitching / myoclonic	33	/	22.6% (33/146)
(2) EEG findings		(No EEG: 11)	
Seizure or seizure-like activity *	43	/	29.5% (43/146)
Seizure activity	6		
Epileptiform Activity	36		
NCSE	1		
Encephalopathy only	86	/	58.9% (86/146)
Normal	6	/	4.1% (6/146)
**3. Infection**			
(1) Pneumonia *	107	39	73.3% (107/146)
(2) UTI *	101	45	69.2% (101/146)
(3) C. Difficile infection *	9	68 (Not tested: 69)	6.2% (9/146)
**4. Metabolic abnormality ^†^—Sodium dysregulation**			
(1) SIADH *	7	139	4.8% (7/146)
(2) Cerebral salt wasting *	2	144	1.4% (2/146)
(3) Diabetes insipidus *	10	136	6.8% (10/146)
**5. Neuroendocrine**			
(1) Panhypopituitary *	3	143	2.1% (3/146)
(2) Low testosterone in male * (*N* = 108)	18	90	16.7% (18/108)
(3) Hypothyroidism *	12	134	8.2% (12/146)
(4) Central adrenal insufficiency *	3	143	2.1% (3/146)

* Considered as one independent condition used for analysis of the total conditions and their concurrency (*N* = 12 in this table). ^†^ Other metabolic abnormalities, including but not limited to potassium/calcium dysregulation, increased creatinine clearance, abnormal liver function panel, hyperglycemia, were seen in almost all patients most commonly transiently. Hyperammonemia was only tested in a very limited amount of patients as its clinical significance is unclear. VPS: ventriculoperitoneal shunt; CT: computed tomography; EEG: electroencephalography; NCSE: non-convulsive status epilepticus; UTI: urinary tract infection; SIADH: syndrome of inappropriate antidiuretic hormone secretion.

**Table 3:** Common neurologic complications and comorbidities that can be confounders or mimics of DoC.

Conditions ^‡^	Number of Cases (*N* = 146, Unless Noted Otherwise)			Incidence Rate
	Yes	Suspected	No	
1. Spasticity *^,†^	139	/	7	95.2% (139/146)
Affected 4 limbs	77	/	/	52.7% (77/146)
**2. Critical illness neuropathy/myopathy** *	9 (EMG confirmed)	8 (no EMG)	129 (EMG ruled out: 12)	Suspected 6.2% (9/146)
**3. Apraxia suspected among those emerged** * (*N* = 85)	/	57	28	Suspected 67.1% (57/85)
**4. Aphasia suspected among those emerged** * (*N* = 85)	/	15	70	Suspected 17.6% (15/85)
**5. Cortical visual impairment** * Neuro-optometry consulted on 93 patients	28 (bilateral 22)	3	115	19.2% (28/146)
**6. Bilateral hearing loss** *	3	/	143	2.1% (3/146)
**7. Locked-in syndrome** *	/	1	145	Suspected 0.7% (1/146)
**8. Catatonia** *	6	41	99	4.1% (6/146)
**9. Akinetic mutism** *	/	4	142	Suspected 2.7% (4/146)

* Considered as one independent condition used for analysis of the total conditions and their concurrency (*N* = 9 in this table). ^†^ For further details, please see Reference [7]. ^‡^ Confounders are conditions affecting the assessment of the level of consciousness (Condition 1–6); mimics are conditions with manifestations similar to but clinically distinct from DoC so they can be mistaken as DoC (Condition 7–9); both of them do not typically affect the level of consciousness.

**Table 4:** Other common neurological and non-neurological complications and comorbidities.

Conditions	Number of Cases (*N* = 146, Unless Noted Otherwise)			Incidence Rate
	Yes	Suspected	No	
**Neurological conditions**				
**1. Paroxysmal sympathetic hyperactivity** *	70	13	63	47.9% (70/146)
**2. Pain**				
(1) Subjectively reported among those emerged * (*N* = 85)	64	10	11	75.3% (64/85)
(2) Confirmed or suspected behaviors of pain in the full cohort	102	/	44	69.9% (102/146)
**3. Complex regional pain syndrome** *	9 (bone scan confirmed)	4 (no bone scan)	133 (bone scan ruled out: 4)	6.2% (9/146)
**4. Spinal cord injury** * (traumatic *N* = 92)	4	/	88	4.3% (4/92)
**Non-neurological conditions**				
**1. Musculoskeletal**				
(1) Heterotopic ossification *	23	/	123	15.8% (23/146)
(2) Spine fracture * (traumatic *N* = 92)	17 (Cervical: 14; thoracic: 4; lumbar: 2)		75	18.5% (17/92)
**2. Respiratory (airway)**				
(1) Tracheotomy status *	143	/	3	97.9% (143/146)
(2) Subglottic stenosis s/p tracheotomy * (*N* = 143) ENT consulted on 78 patients	7	/	139	4.9% (7/143)
**3. Gastrointestinal**				
(1) Ileus *	28	/	118	19.2% (28/146)
(2) Small bowel obstruction *	1	/	145	0.7% (1/146)
(3) Feeding tube status *	146	/	0	100.0% (146/146)
**4. Genitourinary**				
(1) Nephrolithiasis *	23 (CT or renal US: 78)		123	15.8% (23/146)
(2) Hydronephrosis *	2 (CT or renal US: 78)		144	1.4% (2/146)
**5. Ophthalmologic**				
(1) Filamentary keratitis * Neuro-optometry consulted on 93 patients	38	/	108	26.0% (38/146)
**6. Integumentary**				
(1) Pressure ulcer *	103	/	43	70.5% (103/146)
(2) Ingrown nails *	13	/	133	8.9% (13/146)
**7. Venous thromboembolism events**				
(1) Pulmonary embolism (PE) *	12	/	49 (Not tested: 85)	8.2% (12/146)
(2) Deep venous thrombosis (DVT) *	38	/	108	26.0% (38/146)
(3) Both PE and DVT	9	/	137	6.2% (9/146)
**8. Neurovascular injury** (traumatic *N* = 92)				
(1) Vertebral artery dissection *	5	/	87	5.4% (5/92)
(2) Carotid artery dissection *	10	/	82	10.9% (10/92)
(3) Traumatic neurovascular aneurysm/pseudoaneurysm *	4	/	88	4.3% (4/92)
(4) Carotid cavernous fistula *	1	/	91	1.1% (1/92)

* Considered as one independent condition used for analysis of the total conditions and their concurrency (*N* = 22 in this table).

## Data Availability

Anonymized data can be made available on reasonable request.

## Supplementary Materials

The following are available online at https://www.mdpi.com/article/10.3390/brainsci11050608/s1, Table S1: The detailed chart search, review and data collection strategy.
