# Supplementary material for: Many Faces of the Hidden Souls: Medical and Neurological Complications and Comorbidities in Disorders of Consciousness
Source: Brain Sci. 2021 May 10;11(5):608. doi: 10.3390/brainsci11050608 (PMC8151666; doi:10.3390/brainsci11050608)
Supplement: Supplementary file 1 [file brainsci-11-00608-s001.zip › brainsci-1192632-supplementary.pdf]

Supplement Table 1. The detailed chart search, review and data collection strategy

---

Reversible causes:

1. Hydrocephalus status

(1) Hydrocephalus s/p VPS

>> “yes” if documented VPS placement at any point since the initial brain injury; otherwise, “no”

>> Among the patients who had a VPS, “yes” if having confirmed “shunt malfunction” by neurosurgery recommending revision or replacement, or documented VPS revision/replacement; otherwise “no”

(2) CT head findings

>> “hydrocephalus” if documented hydrocephalus in the radiographic report; “ventriculomegaly” can be described as “ventricular dilatation”, “ex vacuo dilatation”, “prominence of the ventricles”, “increase in ventricle size”, etc, in the radiographic report; “No” if documented no hydrocephalus or ventriculomegaly in the report; otherwise, no CT head

2. Seizure

(1) Physical observations

>> 1) Documented history from prior hospitalization in History and Physical; 2) Tonic-clonic manifestation during inpatient rehabilitation stay in progress notes and discharge summary; 3) Limb or facial twitching / myoclonic manifestations during inpatient rehabilitation stay in progress notes and discharge summary; 4) No documented concerning physical findings

(2) EEG findings

>> 1) Seizure or seizure-like activity, including seizure activity, epileptiform activity, non-convulsive status epilepticus; 2) Encephalopathy, without epileptiform activity; 3) Normal; 4) No EEG

3. Infection

(1) Pneumonia

>> Using the key word “pneumonia”; “yes” if clinical concern was expressed, chest x-ray and/or respiratory culture showed evidence, and treatments were initiated; otherwise, “no”

(2) Urinary tract infection (UTI)

>> Using the key word “UTI” and “urinary tract”; “yes” if clinical concern was expressed, urine analysis and/or urine culture showed evidence, and treatments were initiated; otherwise, “no”

(3) C. Difficile infection

>> Checking the results panel; “positive” if any one of the C. difficile DNA tests was positive; “negative” if all the C. difficile DNA tests were negative; otherwise, “not tested”

4. Metabolic abnormality - Sodium dysregulation
(1) SIADH*
>> Using the key word "SIADH"; "yes" if clinical concern was expressed and treatments were initiated; otherwise, "no"
(2) Cerebral salt wasting*
>> Using the key word "cerebral salt wasting"; "yes" if clinical concern was expressed and treatments were initiated; otherwise, "no"
(3) Diabetes insipidus*
>> Using the key word "diabetes insipidus"; "yes" if diagnosed clinically and DDAVP was initiated; otherwise, "no"

5. Neuroendocrine
(1) Panhypopituitary*
>> Using the key word "hypopituitarism"; "yes" if it's indicated per lab results, and replacement treatments for hypogonadism, hypothyroidism and adrenal insufficiency were initiated; endocrinology was typically consulted for management; otherwise, "no"
(2) Low testosterone in male patients* (N = 108)
>> Using the key word "hypogonadism" and "low testosterone"; "yes" if it's indicated per lab results, and replacement treatment was initiated; or when patient has been on replacement treatment; otherwise, "no"
(3) Hypothyroidism*
>> Using the key word "hypothyroidism"; "yes" if it's indicated per lab results, and replacement treatment was initiated; or when a history of hypothyroidism was mentioned since the initial brain injury and the patient has been on replacement treatment; otherwise, "no"
** Subclinical hypothyroidism and history of hypothyroidism prior to the initial brain injury were not counted as an incidence
(4) Central adrenal insufficiency*
>> Using the key word "adrenal insufficiency"; "yes" if it's indicated per lab results, and replacement treatment was initiated; otherwise, "no"

Confounders and mimics:

1. Spasticity*
>> Defined as MAS = 1 or greater in any one of limb muscles per PT/OT notes[7]
>> Spasticity identified in any one of the muscles in each limb was considered affecting the limb; 4-limb spasticity was counted[7]

2. Critical illness neuropathy/myopathy*
>> Using the key word "critical illness"; "suspected" if clinical concern was expressed; "confirmed" if EMG was confirmatory; "ruled out" if EMG was negative; otherwise, "no" if no clinical concern was documented.

3. Apraxia suspected among those emerged* (N = 85)
>> Using the key word "apraxia"; "suspected" if clinical suspicion of oral or limb apraxia was found and expressed in the PT and/or OT and/or SLP and/or neuropsychology notes; "no" if no clinical concern was documented.

4. Aphasia suspected among those emerged* (N = 85)

>> Using the key word “aphasia” and “aphasic”; “suspected” if clinical suspicion was expressed by qualified services (SLP and neuropsychologist); “no” if no clinical concern was documented.

5. Cortical visual impairment*

>> Using the key word “cortical visual impairment”; “yes” if visual impairment was identified in the Function Vision Assessment notes provided by neuro-optometrists, which was determined most likely related to cortical injury and with a visual acuity of 20/200 or worse in either eye; “questionable” if having difficulty determining due to patients’ level of cooperation, however, it was suspected; otherwise, “no”

6. Bilateral hearing loss*

>> Using the key word “deaf”, “deafness”, and “hearing”; “yes” if clinical evidence was found and documented (e.g. by brainstem auditory evoked potential, reported by family, patient unable to follow verbal commands but written commands); “no” if no clinical concern was documented.

7. Lock-in syndrome*

>> Using the key word “lock in”; “suspected” if clinical suspicion was expressed by qualified services (e.g. therapists, neuropsychologists) with appropriate lesion in brainstem; “no” if no clinical concern was documented.

8. Catatonia*

>> Using the key word “catatonia”; “yes” if clinical suspicion was raised and the patient improved with lorazepam; “suspected” if clinical suspicion was expressed by qualified services (e.g. therapists, neuropsychologists); “no” if no clinical concern was documented.

9. Akinetic mutism*

>> Using the key word “akinetic mutism”; “suspected” if clinical suspicion was expressed by qualified services (e.g. therapists, neuropsychologists), with appropriate lesions in the brain (e.g. bilateral medial frontal lesions, basal ganglia thalamofrontal lesions); “no” if no clinical concern was documented.

Other neurological conditions:

1. Paroxysmal sympathetic hyperactivity (PSH)*

>> Using the key word “storming”, “paroxysmal sympathetic hyperactivity”; “yes” if 1) admitted on medications to control PSH which were deemed necessary and continued, 2) consistent signs and symptoms concerning for PSH/storming which required active medical attention and specific medication use; “suspected” if signs and symptoms similar to PSH/storming, without targeted treatments, or considered as a non-primary differential diagnosis; otherwise, “no”

2. Pain*
(1) Subjectively reported pain among those emerged (N = 85)
>> Using the key word “reported pain”; “yes” if a patient reported having pain during daily life and therapy sessions, and at least one source can be identified by the patient; “no” if no pain reported or documented
(2) Confirmed or suspected behaviors of pain in the full cohort
>> Using the key word “pain”; “yes” if a patient reported having pain and at least one source can be identified by the patient, or facial grimacing and/or moaning and/or discomfort was noted during therapy sessions or reported by nursing staffs or family members; “no” if no evidence of pain was reported or documented

3. Complex regional pain syndrome (CRPS)*
>> Using the key word “CRPS”; “suspected” if clinical concern was expressed, further work-up was recommended and/or tentative treatment was initiated; “confirmed” if confirmed diagnosis with triple phase bone scan; “ruled out” if triple phase bone scan didn’t support the diagnosis; “no” if no clinical concerns of CRPS

4. Spinal cord injury (SCI) in traumatic cases* (N = 92)
>> Using the key word “spinal cord” and “SCI”; “yes” if clinical concern was expressed, a SCI specialist was consulted and confirmed the diagnosis; “suspected” if clinical concern was expressed, but not finally confirmed; otherwise, “no”

Other non-neurological conditions:

1. Musculoskeletal
(1) Heterotopic ossification (HO)*
>> Using the key word “heterotopic ossification” and “HO”; “yes” if clinical concern was expressed, which was confirmed by imaging evidence (xray or CT or bone scan), and treatments were initiated; otherwise, “no”
(2) Spine fracture in traumatic cases* (N = 92)
>> Using the key word “spine fracture”; “yes” if identified on radiographic studies, including fractures in the body, pars, and facet joints, excluding spinous and transverse process fractures; otherwise, “no” if not mentioned or no such fractures

2. Respiratory (airway)
(1) Tracheotomy status*
>> Using the key word “trach”; “yes” if tracheotomy was performed or documented as a history; otherwise, “no” if it was not performed or documented
(2) Subglottic stenosis in patients s/p tracheotomy* (N = 143)
>> Using the key word “subglottic stenosis” and “suprastomal stenosis”; “yes” if the condition was diagnosed by ENT and documented in the consult note; otherwise, “no”

3. Gastrointestinal

(1) Ileus*

>> Using the key word “ileus”; “yes” if clinical concern was expressed, which was supported by radiographic evidence of “ileus”, “partial bowel obstruction”, or “hypomotility”, and medical management was initiated mentioned in the progress notes; otherwise, “no”

(2) Small bowel obstruction (SBO)*

>> Using the key word “small bowel obstruction”; “yes” if clinical concern was expressed, which was supported by radiographic evidence of SBO and medical management was initiated mentioned in the progress notes; otherwise, “no”

(3) Feeding tube status*

>> Using the key word “PEG”; “yes” if a gastrostomy tube was placed; otherwise, “no”

4. Genitourinary

(1) Nephrolithiasis*

>> “yes” if identified in any one of the abdominal CT or renal ultrasound reports; otherwise, “no”

>> The imaging studies may or may not be intended for nephrolithiasis

(2) Hydronephrosis*

>> “yes” if identified in any one of the abdominal CT or renal ultrasound reports; otherwise, “no”

>> The imaging studies may or may not be intended for hydronephrosis

5. Ophthalmologic

(1) Filamentary keratitis*

>> Using the key word “keratitis”; “yes” if any description of “exposure keratoconjunctivitis”, “filamentary keratitis”, “superficial punctate keratitis” was identified in the Function Vision Assessment notes provided by neuro-optometrists; otherwise, “no”

6. Integumentary

(1) Pressure ulcer*

>> “yes” if any degree of ulcers was documented in the wound care notes, including stage I-IV, unstageable wounds, deep tissue pressure injury, partial thickness tissue injury or full thickness tissue injury; otherwise, “no”

(2) Ingrown nails*

>> “yes” if the podiatrist was consulted and documented it in the consult note; otherwise, “no”

7. Venous thromboembolism events

(1) Pulmonary embolism (PE)*

>> Using the key word “pulmonary embolism”; “positive” if identified on the CTA PE reports; “history” if patient was found to have PE at outside hospital prior to the rehab admission; “negative” if ruled out on the CTA PE reports; otherwise, “not tested”

(2) Deep venous thrombosis (DVT)*

>> Using the key word “DVT”; “yes” if it’s mentioned in the note as a previous history or identified on the Doppler; “no” if not mentioned or not identified

(3) Both PE and DVT

>> “yes” if having “positive” or “history” on PE and “yes” on DVT; “no” if no combination of the scenarios

8. Neurovascular injury in traumatic cases (N = 92)

(1) Vertebral artery dissection*

>> Using the key word “vertebral artery”; “yes” if the injury to the artery in CTA head/neck and/or angiogram was identified (however, sometimes it may be hard to differentiate grade I injury from vasospasm), or reported in History & Physical as a confirmed history from outside hospital without imaging studies in our system; “no” if no such findings or not mentioned

(2) Carotid artery dissection*

>> Using the key word “carotid artery”; “yes” and “no” criteria same as the above

(3) Traumatic neurovascular aneurysm/pseudoaneurysm*

>> Using the key word “aneurysm”; “yes” if aneurysm or pseudoaneurysm was identified in CTA head/neck and/or angiogram, or reported in History & Physical as a confirmed history from outside hospital without imaging studies in our system; “no” if no such findings or not mentioned

(4) Carotid cavernous fistula*

>> Using the key word “carotid cavernous fistula”; “yes” if it was identified in CTA head/neck and/or angiogram, or reported in History & Physical as a confirmed history from outside hospital without imaging studies in our system; “no” if no such findings or not mentioned

---

VPS: ventriculoperitoneal shunt; CT: computed tomography; EEG: electroencephalography; SIADH: syndrome of inappropriate antidiuretic hormone secretion; DDAVP: desmopressin; MAS: Modified Ashworth Scale; PT: physical therapy; OT: occupational therapy; SLP: speech-language pathology; CTA: computed tomography angiography
